# The implementation of exercise therapy within hospital-based mental healthcare: Delphi study

**DOI:** 10.1192/bjo.2024.717

**Published:** 2024-08-15

**Authors:** Caleb McMahen, Kemi Wright, Ben Jackson, Robert Stanton, Oscar Lederman, Grace McKeon, Simon Rosenbaum, Bonnie Furzer

**Affiliations:** School of Human Sciences (Exercise and Sport Science), University of Western Australia, Perth, Western Australia, Australia; and Fremantle Hospital Mental Health Service, South Metropolitan Health Service, Perth, Western Australia, Australia; School of Health Sciences, University of New South Wales, Sydney, New South Wales, Australia; School of Human Sciences (Exercise and Sport Science), University of Western Australia, Perth, Western Australia, Australia; and Telethon Kids Institute, Perth, Western Australia, Australia; School of Health, Medical, and Applied Sciences, Central Queensland University, Rockhampton, Queensland, Australia; School of Health Sciences, University of New South Wales, Sydney, New South Wales, Australia; and School of Sports Exercise and Rehabilitation, University of Technology Sydney, Sydney, New South Wales, Australia; School of Population Health, University of New South Wales, Sydney, New South Wales, Australia; and Discipline of Psychiatry and Mental Health, University of New South Wales, Sydney, New South Wales, Australia; School of Health Sciences, University of New South Wales, Sydney, New South Wales, Australia; and Discipline of Psychiatry and Mental Health, University of New South Wales, Sydney, New South Wales, Australia; School of Human Sciences (Exercise and Sport Science), University of Western Australia, Perth, Western Australia, Australia; Fremantle Hospital Mental Health Service, South Metropolitan Health Service, Perth, Western Australia, Australia; and Telethon Kids Institute, Perth, Western Australia, Australia

**Keywords:** Exercise therapy, severe mental illness, exercise therapy triage, mental health services, rehabilitation

## Abstract

**Background:**

The physical health comorbidities and premature mortality experienced by people with mental illness has led to an increase in exercise services embedded as part of standard care in hospital-based mental health services. Despite the increase in access to exercise services for people experiencing mental illness, there is currently a lack of guidelines on the assessment and triage of patients into exercise therapy.

**Aims:**

To develop guidelines for the pre-exercise screening and health assessment of patients engaged with exercise services in hospital-based mental healthcare and to establish an exercise therapy triage framework for use in hospital-based mental healthcare.

**Method:**

A Delphi technique consisting of two online surveys and two rounds of focus group discussions was used to gain consensus from a multidisciplinary panel of experts.

**Results:**

Consensus was reached on aspects of pre-exercise health screening, health domain assessment, assessment tools representing high-value clinical assessment, and the creation and proposed utilisation of an exercise therapy triage framework within exercise therapy.

**Conclusions:**

This study is the first of its kind to provide guidance on the implementation of exercise therapy within Australian hospital-based mental healthcare. The results provide recommendations for appropriate health assessment and screening of patients in exercise therapy, and provide guidance on the implementation and triage of patients into exercise therapy via a stepped framework to determine (a) the timeliness of exercise therapy required and (b) the level of support required in the delivery of their exercise therapy.

Compared with the general population, individuals with severe mental illness encounter a wide range of negative impacts on their lives that can include poor quality of life, poor physical health, reduced socialisation, socioeconomic disadvantage and a reduced life expectancy.^1,5^ Life expectancy is typically reduced by 10–20 years for those with severe mental illness,^3,6,7^ with a substantial contribution from premature mortality due to physical disease, including cardiovascular disease and metabolic syndrome.^8,9^ Negative lifestyle factors such as nutritional inadequacies, increased sedentary time and reduced physical activity are highly prevalent in people with severe mental illness, which, in combination with the potential cardiometabolic side-effects of psychotropic medication, contributes to the development and exacerbation of physical illness.^10,12^ Given that poor physical health is a key contributor to premature mortality, there is international advocacy for the improvement of physical health outcomes within mental healthcare.^3^

Exercise is a well-established strategy to improve physical health, owing to its protective effect against cardiovascular disease and metabolic syndrome.^13,14^ Given its benefits, there is increasing advocacy for exercise as an important component of multidisciplinary management of severe mental illness, with specialised exercise professionals (e.g. exercise physiologists and physiotherapists) delivering exercise interventions for people with severe mental illness.^3,15^ Despite this advocacy for the inclusion of exercise in multidisciplinary mental healthcare, there are few documented examples where exercise services have been embedded into hospital-based mental healthcare, with the examples available containing substantial heterogeneity in intervention, setting, resources and outcome reporting, contributing to difficulties in developing guidelines for implementation. The lack of implementation guidelines poses a challenge for exercise services in establishing models of care and evaluating outcomes, which can potentially lead to poor service efficiency, mismanagement of resources and an exacerbation of resource demand, all of which can lead to an overall decline in patient outcomes from exercise therapy. The establishment of guidelines for the implementation of exercise therapy in hospital-based mental healthcare may assist services in developing models of care to provide guidance on clinical reporting and the triage of patients into exercise therapy.

A scoping review recently conducted in this area reported inconsistencies in the assessment and health screening of people with severe mental illness for exercise interventions,^16^ and it was noted that expert opinion is required to translate the findings of the review into exercise services. For the current study, we therefore employed a Delphi technique to incorporate expert opinion into developing guidelines for exercise services within hospital-based mental healthcare.

The Delphi technique is a group communication process aiming to achieve convergence of opinions between experts in a field.^17^ The purpose of this Delphi study was to establish consensus between experts in mental healthcare and/or exercise to develop guidelines on the implementation of exercise therapy in hospital-based mental health services. The study had two specific aims. The first aim was to develop consensus on the best practice guidelines for the pre-exercise screening and health assessment of patients engaged with exercise services in hospital-based mental health services. The second aim was to establish an exercise therapy triage framework for hospital-based mental health services, to guide the identification of patients at the greatest need of timely exercise therapy and to assist in determining the relevant supports, skills and expertise required for their exercise therapy.

## Method

This Delphi study was guided by previous literature, with recommendations to design the Delphi (i.e. order of the study, number of participants and number of rounds) based on the unique topic of interest.^17,21^

### Recruitment

The study's recruitment limits were a minimum of 10 and a maximum of 50 participants, to ensure that individual professions (i.e. nursing, allied health, medicine, administrators and academics) were represented and accommodate variance in acceptance and drop-out, while avoiding an overpopulated sample in which consensus becomes more challenging to reach.^17^ An expert panel of experts in the field of mental health and/or exercise (the participants) was identified for recruitment to the initial round of surveys via professional connections or authorship in academic publications related to severe mental illness and exercise, physical activity or physical health. Recruitment was via email and the initial recruitment email was delivered to 46 potential panellists. Participants gave informed consent by completing an electronic participant information and consent form included in the initial email recruitment and survey invitation.

The authors assert that all procedures contributing to this work comply with the ethical standards of the relevant national and institutional committees on human experimentation and with the Helsinki Declaration of 1975, as revised in 2008. All procedures involving human participants/patients were approved by The University of Western Australia Human Research Ethics Committee 2019/RA/4/20/5998.

### Delphi protocol

**Fig. 1:**
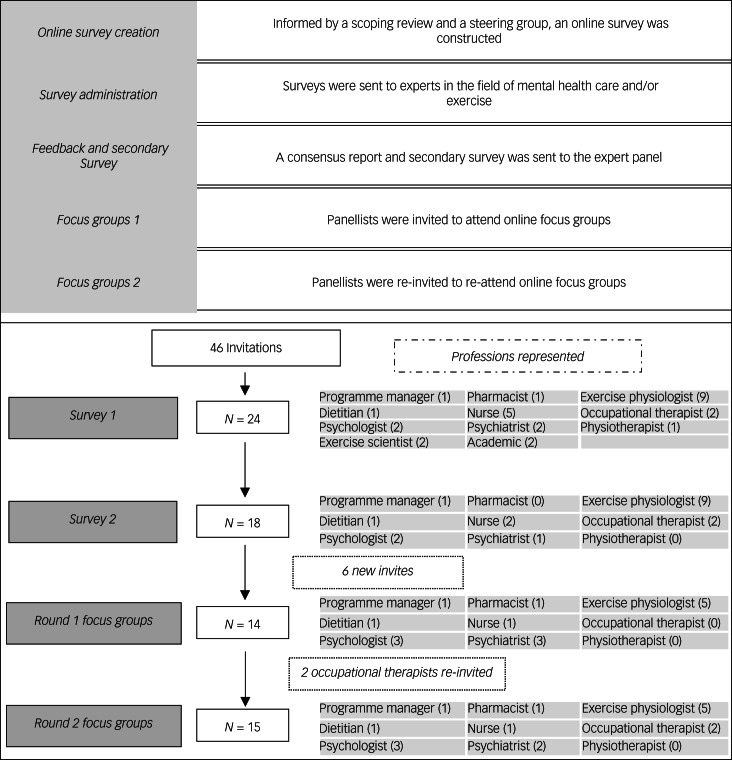
The Delphi study and participant flow.

The optimal number of rounds and structure of each round within a Delphi study is unique to each topic.^19,22^ Our study required a minimum of three rounds to ensure that both phases of the study could take place (i.e. two initial rounds of surveying and at least one round of focus groups) and a maximum of four rounds to establish a discrete end-point. This Delphi study consisted of an online survey hosted on the Qualtrics survey platform (www.qualtrics.com), a consensus report and re-administration of the online survey, and two rounds of focus group discussions. Each round of focus groups consisted of four sessions, with two to six participants in each session, chaired by the lead author (C.M.). The focus groups were conducted online via Microsoft Teams with audio-visual recording. Audio was transcribed by automated software (www.Otter.ai), with author C.M. confirming transcription accuracy. Fig. 1 details the steps in this study.

In the initial survey panellists were asked to provide Likert-scale responses (e.g. strongly agree, agree, neutral, disagree, strongly disagree) to certain statements on exercise therapy and severe mental illness (e.g. ‘Patients participating in exercise therapy should undergo assessments of body composition’). Panellists were then asked to rank individual assessment tools in relation to what they believed reflected best practice assessment in exercise therapy and severe mental illness (e.g. ‘Please rank the body composition tools below in order of what you believe most to least reflects best practice assessment’). In the re-administration of the survey, panellists were informed of the results of the initial survey and asked to respond ‘yes’ or ‘no’ to whether they agreed with each result.

The initial focus group discussions were guided by four main discussion points presented as broad questions, allowing for discussion by the expert panel on these topics. The four topics of discussion were (a) identifying poor physical health in severe mental illness, (b) when mental health professionals currently refer patients to exercise services, (c) the key factors in predicting the physical health decline of patients and (d) the key factors in the triage of patients into exercise therapy.

### Data analysis

The main outcome of this study was the expert panel consensus, with consensus set at a minimum of 75% agreement or disagreement, guided by previous literature.^23^ Analysis of results was conducted using IBM SPSS statistics.^24^ For Likert scales and yes/no response statements, agreement and disagreement was analysed; however, ranking questions were analysed for mean score initially, with the two top ranked responses being presented to the panel to seek consensus. Text entry responses were identified for discussion in the focus groups. For the secondary round of surveying, questions were primarily yes/no responses to the initial results, with a further 75% yes (or no) deemed as receiving consensus.^23^

As the focus group stage of this study employed qualitative methods, an inductive thematic analysis was used to group content by the theme of discussion.^25^ The thematic analysis was reflexive and, following initial thematic analysis, research team meetings were conducted to implement a ‘critical friends’ approach, to clarify themes and guide the creation of an exercise therapy triage framework.^26^ Thematic analysis was conducted at the conclusion of each round of focus groups. On completion of the round one focus groups, panellists were provided a written report of the results and the preliminary triage framework, with the second round of focus groups used to confirm the results and discuss the proposed use of the framework. After the final focus group, all results of consensus and the exercise therapy triage framework were compiled into a report and delivered via email to the panellists to allow for any final disagreements or queries on results.

## Results

Of the 46 individuals initially invited, 24 contributed to this study. Of these, 17 were female (71%) and 7 male (29%), with an average of 9 years’ experience in mental healthcare (s.d. = 7.8 years). Professions varied and participants were able to indicate multiple professions (e.g. an academic could also be a pharmacist). The professions represented at each stage of the study are shown in Fig. 1. Hospital settings were the most frequent service background (83%). Nearly all participants were based in Australia (92%), with one individual in Switzerland (4%) and one in the UK (4%). There was participant attrition throughout each round, with participants of a similar profession replacing them where possible, leaving 15 contributors in the final round (Fig 1). The first round of focus groups consisted of three group sessions and one individual interview averaging 51 min in duration (range 50–52 min), with variation in participant numbers (*n* = 2, 5 and 6 respectively). The second round of focus groups consisted of three group sessions averaging 54 min in duration (range 50–60 min) and similarly varied in participant numbers (*n* = 4, 5 and 6 respectively).

### Pre-exercise screening

Pre-exercise health screening was examined to provide insight into the prevention or management of adverse events in exercise therapy, with all statements related to pre-exercise screening examined in this study receiving consensus (preliminary consensus; secondary consensus). It was determined that patients should undergo a standardised, population-specific pre-exercise screening because of the potential for adverse events related to exercise (preliminary consensus 91.7%; secondary consensus 100%), with exercise professionals best placed to conduct this screening (83.3%; 100%) owing to their knowledge and ability to safely conduct pre-exercise screening in this population (82.5%; 100%). There was consensus that at a minimum, pre-exercise blood pressure (95.5%; 100%) and heart rate (95.5%; 100%), and an adult pre-exercise screening questionnaire (86.4%; 100%) should be part of screening prior to structured exercise therapy.

### Health assessment

Seven statements were related to general health assessment within exercise therapy, with six receiving consensus (preliminary consensus; secondary consensus). The six statements receiving consensus were the need for health assessment beyond pre-exercise screening (preliminary consensus 83.5%; secondary consensus 100%), exercise professionals to conduct physical health assessments (95.8%; 94.4%), physical health assessments conducted by exercise professionals adding value to patient care (100%; 94.4%), mental health assessments conducted by exercise professionals adding value to patient care (83.3%; 83.3%), that exercise professionals not be limited to assessing exercise-specific outcomes (91.3%; 94.4%) and that outcome assessment and monitoring should be more than intake and discharge from programmes (83.3%; 100%). Despite the recognised potential for added value to patient care, there was no consensus for exercise professionals to regularly assess mental health outcomes (62.5%; 72%).

Fourteen statements related to the general assessment of health domains and the scope for exercise professionals to conduct assessment of certain health domains, with results indicated in Table 1. There were then 11 questions on the method or tool of assessment of the associated health domains, with 8 health domains reaching consensus for specific tools that represent appropriate and high-value clinical assessment, and 2 health domains with consensus on general assessment recommendations, with results in Table 2. For the assessment of balance in exercise therapy there were initially no tools endorsed, and on further questioning a non-specific, general comprehensive balance assessment (i.e. static and dynamic) received consensus for use (77.0%). In contrast, cardiorespiratory fitness also initially had no assessment tools endorsed and further discussion did not elicit consensus for a specific cardiorespiratory fitness assessment tool, with the greatest recommendations for submaximal exercise testing (62%), which did not reach consensus levels. Finally, there were no specific assessment tools recommended to use in the assessment of exercise beliefs and attitudes; however, there was consensus for the general assessment of exercise self-efficacy in exercise therapy (78.6%).

**Table 1.** Consensus results for health domains

Health domains to be assessed within exercise therapy	Consensus outcome (preliminary %; secondary %)
Body composition	Yes (81.8; 100.0)
Cardiorespiratory fitness	Yes (90.9; 100.0)
Metabolic blood markers	Yes (81.0; 100.0)
Muscle function	Yes (90.9; 100.0)
Balance	Yes (90.0; 100.0)
Physical function	Yes (81.9; 100.0)
Urine markers	No (44.8; 87.0)
Health domains within the scope of exercise professionals	Consensus outcome (preliminary %; secondary %)
Psychosocial function	Yes (81.9; 100.0)
Physical activity and sedentary behaviour	Yes (95.2; 100.0)
Exercise beliefs and attitudes	Yes (90.5; 100.0)
Sleep	Yes (83.4; 100.0)
Diet	Yes (65.2; 83.0)
Quality of life	Yes (71.4; 88.0)
Disorder specific severity	No (60.0; 59.0)

**Table 2.** Consensus results for assessment tools

Health domain	Assessment tools	Consensus outcome (%)
**Specific tools representing appropriate and high-value clinical assessment**		
Body composition	Body mass index (BMI) Girths (waist and hip circumference)	Yes (94.0)
Metabolic blood markers	Lipid profile Blood glucose level	Yes (95.0)
Muscle function	Handgrip dynamometry 30 s repetition tests	Yes (88.0)
Quality of life	World Health Organization – Quality of Life – BREF Quality of Life – Enjoyment and Satisfaction Questionnaire	Yes (100.0)
Physical activity and sedentary behaviour	Simple Physical Activity Questionnaire International Physical Activity Questionnaire	Yes (100.0)
Diet	3-day food recall Food diary	Yes (87.0)
Psychosocial function	Global Assessment of Functioning Social and Occupational Functioning Scale	Yes (92.0)
Sleep	Simple Physical Activity Questionnaire Pittsburgh Sleep Quality Index	Yes (100.0)
**General assessment tools representing appropriate and high-value clinical assessment**		
Balance	A comprehensive balance assessment	Yes (77.0)
Exercise beliefs and attitudes	Assessment of exercise self-efficacy	Yes (78.6)
Cardiorespiratory fitness	A submaximal exercise test 6-minute walk test	No (62.0) (31.0)

### Focus groups

During the focus groups, panellists were encouraged to offer their opinions throughout on topics of discussion; on conclusion and analysis of the round one focus groups any outcomes agreed upon or lacking disagreement from the panel were deemed to have received consensus. The thematic analysis conducted at the conclusion of the round one focus groups generated major themes for topics one and two, and major themes and sub-themes for topics three and four.

For topic one, discussion varied in identifying poor physical health in severe mental illness; however, there was consistency in the major themes of ‘visual observation’, ‘patient negative lifestyle factors’, ‘change in lifestyle factors’ and ‘physiological markers’. The second topic – when mental health professionals currently refer patients to exercise services – related largely to the presentations that require specialist exercise input, with major themes of referrals in the presence of ‘increased clinical complexity or comorbidity’, ‘patients requiring high levels of support to become physically active’ and that exercise service referrals should be for ‘preventing physical health decline’. Panellists also considered the ‘patient's willingness to the referral’ before referring to exercise services.

**Fig. 2:**
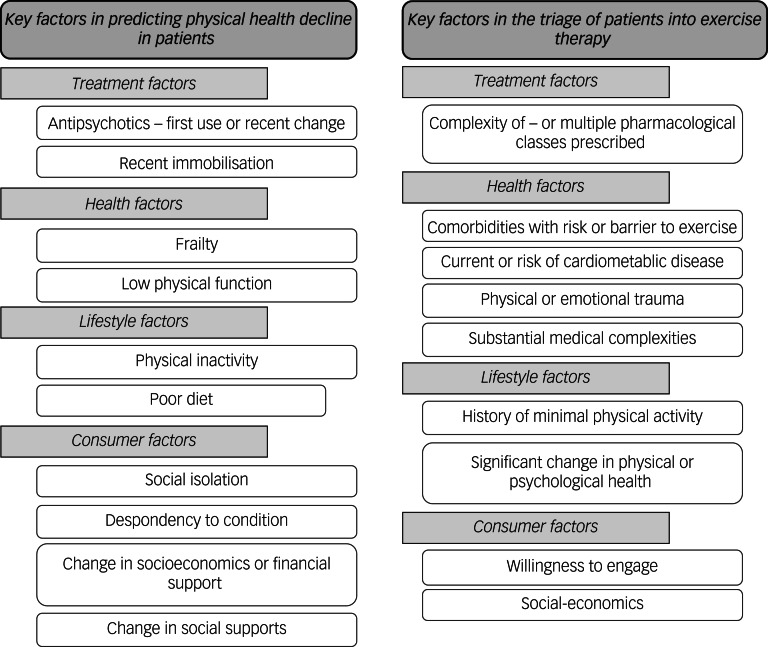
Themes and sub-themes of the key factors in predicting physical health decline in patients and the triage of patients into exercise therapy.

Topics three and four – the key factors in predicting physical health decline in patients and the key factors in the triage of patients into exercise therapy – generated substantial discussion, as this was a major focus of this research, with themes and sub-themes generated. Both topics had the consistent major themes of ‘treatment factors’, ‘health factors’, ‘lifestyle factors’ and ‘consumer [patient] factors’, with sub-themes within these themes. The major themes and sub-themes from topics three and four of the first round of the focus groups are shown in Fig. 2.

### Secondary focus groups

In the second round of focus groups, panellists were invited to discuss the results of the first round. Consensus was reached on all aspects of the outcomes established in the first round, including agreement on the results of the thematic analysis and creation of the exercise therapy triage framework along with the proposed utilisation of the exercise therapy triage framework. There were slight disagreements to certain terminology raised during the secondary focus groups, with suggestions to reword and clarify some factors in the exercise therapy triage framework, with all suggestions endorsed by the expert panel and implemented by the research team. Examples of suggested changes included changing ‘antipsychotic use’ to more broadly ‘psychotropic medications’, reclassifying comorbidities that are barriers or risks to exercise into separate factors for physical or psychiatric comorbidities and replacing ‘social-economic’ with specific items relating to access to resources.

The conclusion of both rounds of the focus groups culminated in three major outcomes from this study relating to the triage of patients into exercise therapy in hospital-based mental healthcare. First, patients should be triaged into exercise services, utilising a triage process to enhance the ability of exercise services to provide care and to inform the appropriate therapy for individual patients. Second, mental health professionals already apply an informal triage to exercise therapy by preferentially referring patients based on the needs and suitability of the patient, the perceived difficulty for the patient to become physically active independently and the willingness of the patient to be referred to and engage with an exercise service. Last, and most significant, the panel provided guidance on how to triage patients into exercise therapy within hospital-based mental healthcare by using two tiers of patient-related factors to determine the timeliness and type of exercise therapy for each patient.

## Discussion

This study achieved the outlined aims by developing consensus on best practice guidelines for pre-exercise screening and health assessment for exercise therapy in mental health services and establishing an exercise therapy triage framework to guide exercise service models of care.

### Health screening and assessment

Supplementary Material 1 provides a list of the recommendations for pre-exercise health screening and health assessment relevant to exercise therapy within hospital-based mental health services, available at https://doi.org/10.1192/bjo.2024.717. Results of this study demonstrate the need to conduct such pre-exercise health screening, owing to the prevalence of comorbidities seen in this population and the risk of adverse events during exercise therapy, and that exercise professionals are best placed to conduct pre-exercise health screening. This research outlines that at a minimum, pre-exercise heart rate and blood pressure should be examined, alongside an adult pre-exercise screening questionnaire. This level of pre-exercise screening is suggested as the minimum requirement, and each exercise service will have unique screening requirements, dependent on patient base and illness severity. The expert panel noted that extensive pre-exercise screening may further increase barriers to exercise, and the balance between adequate health screening and patient burden must be considered.

There is clear recommendation for exercise professionals' scope to carry out assessment beyond exercise-based assessments, with recognition of the value of exercise professionals' involvement in patients’ physical health monitoring. This is particularly evident given the literature underlining that physical health and health behaviours are poorly reported in mental healthcare,^10^ with potential for exercise professionals to fill the gap in health monitoring of patients, potentially leading to greater holistic care. Although the value of assessment of mental health outcomes in exercise therapy by exercise professionals was established, there was no clear consensus on the scope for ongoing assessment by exercise professionals. The expert panel advised caution with respect to exercise professionals monitoring patients’ mental health outcomes, with other members of the multidisciplinary team potentially better placed to conduct structured outcome assessments; however, if assessment is to be conducted by exercise professionals, communication with health professionals is required. It was noted that in the absence of a comprehensive multidisciplinary team, the scope for exercise professionals in assessing mental health outcomes will increase owing to limitations in adequate monitoring by other health professions.

There are clear recommendations on the aspects of physical health requiring ongoing assessment during exercise therapy in mental health services – body composition, cardiorespiratory fitness and metabolic blood markers – and on the tools deemed appropriate and offering high-value clinical assessment of body composition and metabolic blood markers (Supplementary Material 1). That these domains received consensus is unsurprising given the extensive evidence detailing the high rates of adiposity, low cardiorespiratory fitness, worsened blood lipid profile and higher blood glucose levels in severe mental illness, and their contribution to the development of cardiovascular disease and metabolic syndrome.^27,29^ There is additional recommendation to assess muscle and physical function as part of exercise therapy, with specific assessment tools being identified by the expert panel as appropriate and high value (Table 2). It was recommended that balance be assessed, likely owing to the potential for impaired balance with psychotropic polypharmacy;^30^ however, no specific assessment tools received consensus for use from the expert panel.

With respect to the scope for exercise professionals to conduct ongoing assessments of psychosocial function and other lifestyle behaviours (e.g. diet and sleep), the expert panel concluded that there are potentially better placed health professionals (e.g. dietitians) to conduct these assessments. In the absence of specific expertise, these domains should be assessed by exercise professionals if they have adequate time and training. For the assessment of psychosocial function, quality of life and other lifestyle behaviours (e.g. physical activity, diet, sleep, sedentary behaviour) the expert panel reached consensus on appropriate, high-value clinical assessment tools (Table 2).

Despite the results of this study indicating the value of incorporating the pre-exercise screening and assessment items outlined in Supplementary Material 1 as part of exercise therapy, introducing aspects of this health assessment monitoring may not be feasible for all mental health services, considering the additional costs, staff time and expertise required. Consequently, although the results in Supplementary Material 1 outline the expert panel's suggestion of pre-exercise health screening and health assessment within exercise therapy, the implementation of these recommendations will be dictated by factors unique to each mental health service.

Supplementary Material 1 provides a full list of recommended pre-exercise screening, health domain assessment and appropriate assessment tools for use in exercise therapy within hospital-based mental health care.

### Exercise therapy triage

**Fig. 3:**
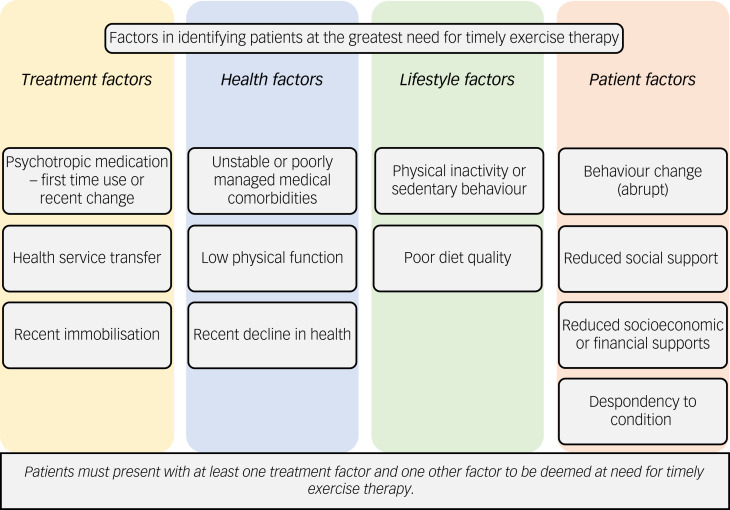
Factors in identifying patients at the greatest need for timely exercise therapy.

As regards exercise therapy triage, this research outlines two independent frameworks to guide exercise services on who will benefit most from immediate exercise therapy (i.e. timeliness) and what type of therapy they are suited to (i.e. support required). The first tier of the triage framework, identifying the patients at the greatest need for timely exercise therapy, is informed by the heightened risk of decline in physical health and greatest potential benefit from timely exercise therapy (Fig. 3). This tier of the framework is to be used by clinicians to identify the patient factors that may play a role in exacerbating decline in physical health and therefore the patients that are at great need of timely exercise therapy. For an example assessment protocol to utilise in the determination of the first tier of the triage framework see Supplementary Material 2.

**Fig. 4:**
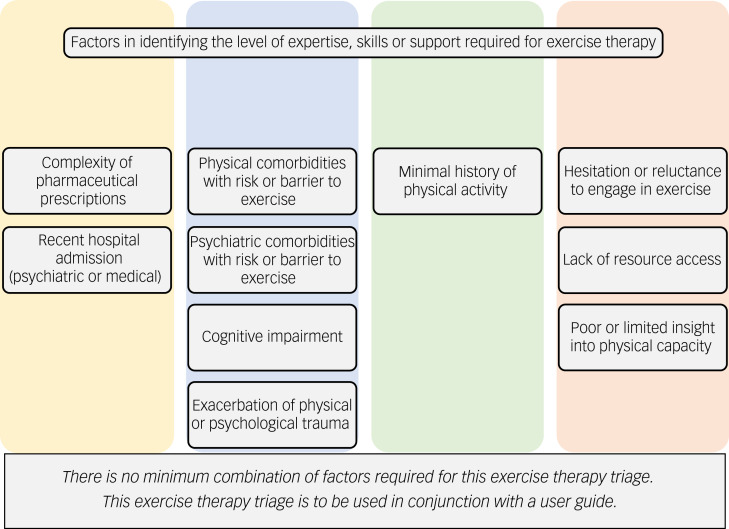
Factors in identifying the level of expertise, skills or support required for exercise therapy.

The second tier of the triage framework, identifying the level of expertise, skills or support required in exercise therapy, assists in determining the most suitable mode of exercise therapy for individual patients (Fig. 4). Determining the most suitable type of exercise therapy should consider the level of expertise, skill or support required for the patient and should be done by identifying the patient factors outlined in the second tier of the triage framework that may indicate whether a patient has high or low levels of support needs in exercise therapy. Some factors are subjective (e.g. history of minimal physical activity) and clinician discretion is advised in determining the magnitude of the factors present for a patient, particularly considering that services may have patients present with multiple factors. For an example assessment protocol to use in the determination of the second tier of the triage framework see Supplementary Material 3.

Although this exercise triage framework is designed for use by exercise services in hospital-based mental healthcare, it has the potential for use by other members of the multidisciplinary team. The framework can be used to assist in identifying patients to refer (or refer more promptly) to exercise services (Fig. 3) or which patients may not require specialised exercise services owing to their low support needs (Fig. 4). An important consideration from the expert panel is the potential ethical dilemma if the triage framework led to patients having care withheld, particularly considering the benefit of exercise therapy to patients beyond physical health improvements. To mitigate against this, the exercise therapy triage framework needs to allow flexibility to consider provision of exercise therapy to patients not identified by the first tier of triage framework. Furthermore, factors within the exercise therapy triage are not equally weighted in terms of impact on individual patients’ physical health (e.g. total number does not reflect a proportional increase in health risk), and therefore clinicians should use their expertise and judgement when considering those presenting with multiple factors.

The exercise therapy triage framework is complemented by a user guide including definition of terms, quantifying factors, example assessment sheets and examples of use. The user guide includes a resource created in conjunction with the expert panel categorising the potential metabolic impacts of certain psychotropic medication.

### Translation to clinical practice

The adoption of the pre-exercise screening and health outcome assessment recommendations for exercise therapy from this research may improve exercise services’ management of adverse events and reporting of patient outcomes. Implementing the exercise therapy triage framework may improve exercise services’ allocation of resources, by more effectively identifying patients who require priority for exercise therapy, and assist in determining the type of therapy suited to each individual. In combination, adopting the outcomes of this research may lead to improved reporting of patient outcomes, improved exercise service efficiency, improved level of care provided to patients and ultimately improved patient outcomes from engaging in exercise therapy.

### Strengths and limitations

This research involved individuals with extensive and diverse experience in mental health services, providing a multidisciplinary view of mental healthcare. However, given the research design there are some limitations that are worth considering. First, there was potential for opinions to be overshadowed by dominant individuals in the focus group setting; however, the group chair (C.M.) aimed to minimise this at each phase, including different group allocations in the second round of focus groups. Second, the recruited panellists were likely to value exercise in the management of mental illness, owing to their acceptance of the invitation to participate, and therefore may have an inherent bias towards exercise and its use in mental healthcare. Third, this research primarily relates to severe mental illness within hospital mental health services in which increasing physical activity and improving health behaviours are the targets. Psychiatric comorbidities, in which the goals of exercise therapy may not be to increase physical activity (e.g. feeding and eating disorders), were briefly discussed; however, the results of this research may not be applicable to those patients, and clinicians should use their discretion in determining whether the exercise therapy triage framework is appropriate for their unique mental health services. Fourth, although this study incorporated discussion of wider mental health services, the expert panel were largely based in Australia and therefore the results may have reduced applicability to regions outside of Australia. Fifth, exercise professionals were the most represented profession, owing to the scope of the study, and the study may have benefited from an increased representation from other health professions, alongside a greater physiotherapy representation from among the exercise professional cohort. Finally, this study related to health professionals’ perspectives; however, patients’ perspectives are increasingly incorporated into mental health research to increase the successful translation of research outcomes into patient care.^31,32^ This study might have been strengthened by including patient perspectives on exercise therapy implementation, as this might have identified aspects of service design affecting engagement and uptake of exercise therapy that may not have been considered by the participants.

## Supporting information

McMahen et al. supplementary material 1 McMahen et al. supplementary material

McMahen et al. supplementary material 2 McMahen et al. supplementary material

## Data Availability

Data supporting this study are available from the corresponding author.
