# Supplementary material for: The implementation of exercise therapy within hospital-based mental healthcare: Delphi study
Source: BJPsych Open. 2024 Aug 15;10(5):e147. doi: 10.1192/bjo.2024.717 (PMC11698169; doi:10.1192/bjo.2024.717)
Supplement: McMahen et al. supplementary material 1 — McMahen et al. supplementary material [file S2056472424007178sup001.docx]

**Check (×) each box if the patient has this present**

Identifying Patients at the Greatest Need for Timely Exercise Therapy

**Treatment Factors ( ≥ 1)**

Have they had a recent change or first-time use of psychotropic medication?

Have they recently changed between health services (e.g., inpatient to outpatient)?

Recent immobilisation (e.g., injury or hospitalisation)

**≥ 1 of these factors?**

≥ 1 factor from the below categories

**Health Factors**

Unstable or poorly managed medical comorbidities

Recent decline in health status

Low level of physical function

**Lifestyle Factors**

Physical inactivity or sedentary behaviour Poor diet quality

**Consumer factors**

Abrupt change in behaviour

Reduced social support Reduced financial supports Despondent to condition

**≥ 1 of these factors? Total**

≥ 1 treatment and ≥ 1 other factor

**Supplementary Material 2. Exercise Therapy Triage Framework Example Assessment Tier 2.**

**Check (×) each box if the patient has this present**

Identifying Level of Expertise, Skills, or Support Required for Exercise Therapy

**Treatment Factors**

Do they have multiple (or complex) different pharmacological classes prescribed?

**List**:

A recent hospital (medical or psychiatric) admission

**Recency:** **Duration:** **Reason:**

**Health Factors**

Physical comorbidities with risk/barrier to exercise therapy.

Psychiatric comorbidities with risk/barrier to exercise therapy.

Cognitive impairment Exacerbation of psychological or physical trauma

**Lifestyle Factors**

Minimal history of physical activity

**Consumer factors**

Reluctancy to engage in exercise Lack of resource access

Limited insight into physical capacity

**Total**
