# Supplementary material for: The implementation of exercise therapy within hospital-based mental healthcare: Delphi study
Source: BJPsych Open. 2024 Aug 15;10(5):e147. doi: 10.1192/bjo.2024.717 (PMC11698169; doi:10.1192/bjo.2024.717)
Supplement: McMahen et al. supplementary material 2 — McMahen et al. supplementary material [file S2056472424007178sup002.docx]

| Pre-exercise Health Screening for Exercise Therapy | | |
| --- | --- | --- |
| An Adult Pre-exercise Screening Questionnaire  E.g., Adult Pre-Exercise Screening System (Exercise & Sport Science Australia et al., 2019) | Resting Blood Pressure | Resting Heart Rate |
| Health Assessment for Exercise Therapy | | |
| *Health Domains to Assess in Exercise Therapy* | ***Appropriate and High Value Clinical Assessment Tools*** | |
| *Body Composition* | *Body Mass Index*  *Waist & Hip Circumference* | |
| *Metabolic Blood Markers* | *Lipid Profile*  *Blood Glucose* | |
| *Muscle Function* | *Handgrip Dynamometry*  *30s Repetition Tests* | |
| *Psychosocial Function* | *Global Assessment of Functioning*  *Social and Occupational Functioning Scale* | |
| *Physical Activity and Sedentary Behaviour* | *Simple Physical Activity Questionnaire*  *International Physical Activity Questionnaire* | |
| *Sleep* | *Simple Physical Activity Questionnaire*  *Pittsburgh Sleep Quality Index* | |
| *Diet* | *3-day Food Recall*  *Food Diary* | |
| *Quality of Life* | *World Health Organisation Quality of Life – BREF*  *Quality of Life- Enjoyment and Satisfaction Questionnaire* | |
| *Balance* | *Nil Specific Tool Receiving Consensus*   - *Comprehensive Balance Assessment* | |
| *Exercise Beliefs & Attitudes* | *Nil Specific Tool Receiving Consensus*   - *General Exercise Self-Efficacy* | |
| *Cardiorespiratory Fitness* | *Nil Tool Receiving Consensus* | |
| *Physical Function* | *Nil Tool Receiving Consensus* | |

Table 3. Pre-exercise screening and health assessment for exercise therapy in hospital-based mental health services based on consensus results.
